# Parameter Estimation of Dynamic Beer Fermentation Models

**DOI:** 10.3390/foods11223602

**Published:** 2022-11-11

**Authors:** Jesús Miguel Zamudio Lara, Laurent Dewasme, Héctor Hernández Escoto, Alain Vande Wouwer

**Affiliations:** 1 Systèmes, Estimation, Commande et Optimisation, Université de Mons, 7000 Mons, Belgium; 2 Departamento de Ingeniería Química, Universidad de Guanajuato, Guanajuato 36050, Mexico

**Keywords:** parameter estimation, mathematical modeling, beer fermentation, food industry

## Abstract

In this study, two dynamic models of beer fermentation are proposed, and their parameters are estimated using experimental data collected during several batch experiments initiated with different sugar concentrations. Biomass, sugar, ethanol, and vicinal diketone concentrations are measured off-line with an analytical system while two on-line immersed probes deliver temperature, ethanol concentration, and carbon dioxide exhaust rate measurements. Before proceeding to the estimation of the unknown model parameters, a structural identifiability analysis is carried out to investigate the measurement configuration and the kinetic model structure. The model predictive capability is investigated in cross-validation, in view of opening up new perspectives for monitoring and control purposes. For instance, the dynamic model could be used as a predictor in receding-horizon observers and controllers.

## 1. Introduction

Beer is the most consumed alcoholic beverage worldwide and is produced by the fermentation of sugars in the wort by yeasts [1]. The production of beer in 2019 was 1912 million hectoliters (hl), while in 2020, the production reduced to 1820 million hl due to the COVID-19 pandemic [2]. Beer production is a complex biochemical process in which the main ingredients are water, malt (sugar source), yeast, and hops [3]; however, other products can be added, such as fruits, chocolate, and coffee grains, among others.

The fermentation stage is crucial to guarantee good quality beer since it is when all the nutrients, flavor, and odor components are produced, in addition to ethanol. At this stage, yeast is introduced in the wort (broth that is rich in sugars) from the boiling stage at the desired temperature. The main chemical reaction is the conversion of these sugars into ethanol and carbon dioxide, along with biomass growth and heat generation. At the same time, several secondary reactions occur, generating several components at lower concentrations that contribute to the flavor and aroma characteristics.

To enhance the fermentation, several factors, such as yeast pitching rate, dissolved oxygen, batch pressure, and temperature, must be taken care of by the brewers [4]. Among these factors, temperature is important as it helps accelerate the fermentation but needs to remain within controlled bonds to avoid yeast death (above 30 °C), the production of undesirable byproducts, and the growth of bacteria, damaging the final product. Therefore, rigorous control of the temperature inside the fermenter must be exercised to ensure product quality and alleviate variations between batches.

In the brewing industry, time-varying temperature profiles are established along the fermentation process in order to alleviate the above-mentioned potential issues [5]. Looking for the appropriate temperature profile is, however, not an easy task, and experimental determination can be time-consuming. Model-based optimization is, therefore, an appealing alternative. Dynamic models can be useful not only to optimize the operating conditions, but also to design state estimators reconstructing online non-measured variables or designing controllers to ensure close setpoint tracking [6].

To this end, Gee and Ramirez [7] proposed a detailed model of beer fermentation describing biomass growth and the production of flavor compounds through macroscopic reactions inferred from biological pathways. This model segregates the sugars into glucose, maltose, and maltotriose. The derivation of the corresponding sugar uptake kinetics is, therefore, the center of interest, and the related parameters are assumed to be temperature-dependent. However, this model is unlikely to be applicable for control purposes since it involves variables that require specific monitoring equipment beyond the standards of most fermenters. Andrés-Toro et al. [8], conversely, proposed to segregate the yeast (biomass) into three types: lag, active, and death, while the sugars are considered as a whole. Sugar monitoring, therefore, appears simpler, and the model also takes account of industrial operational characteristics as well as the undesired beer flavor caused by ethanol and byproduct (diacetyl and ethyl acetate) formation. The main drawback of this model is the difficulty in obtaining accurate biomass data. Trelea et al. [9] developed what can be considered as, so far, the most practical model, where only three states are considered: the dissolved carbon dioxide concentration used as an image of the growing biomass, the ethanol concentration, and the sugar concentration. The main advantage of this latter model lies in its practical control-oriented description of the fermentation process, considering variables that can be easily measured and tracked.

The objective of this work is to revisit these classical models, propose a few adaptations, and develop a thorough study of the parameter estimation problem based on a popular fermentation device, e.g., a 30-L stainless-steel Grainfather^®^ fermenter. Two alternative mathematical models are considered, one based on yeast (biomass) and the other on carbon dioxide. The difference between these models is discussed in terms of biological interpretation, bioreactor instrumentation, and data collection (i.e., parameter estimation, model validation, and process control perspectives). As a result, models with good predictive capability are proposed together with their experimental validation.

This paper is organized as follows. The next section describes the experimental setup, while Section 3 presents a review of dynamic models of beer fermentation, together with possible model adaptations required to represent the considered case study. Section 4 develops a structural identifiability analysis based on the software tool Strike Goldd [10]. Section 5 introduces a parameter identification procedure, including parametric sensitivity analysis and model validation. The last section draws the main conclusions of this work and discusses the monitoring and control perspectives.

## 2. Beer Fermentation Experimental Set-Up

The pilot plant consists of a stainless-steel conical fermenter (30 L, Grainfather^®^), which has a built-in sensor to measure the temperature of the liquid content. This sensor is paired to a control system connected to a glycol chiller (Grainfather^®^) to keep the temperature regulated. Ethanol and carbon dioxide concentrations are measured online, respectively, with a tilt^®^ hydrometer and a Plaato^®^ airlock.

The hydrometer is introduced in the wort and keeps floating in a tilted position, measuring the specific gravity which also allows, based on some predetermined correlations, for assessing the percentage of alcohol. The sensor also has an integrated temperature sensor. The airlock consists of four components: a lid, a bubbler, a Tritan, and a smart part (containing the temperature and infrared sensors). This device measures the evenly-sized bubbles of carbon dioxide released by the wort and converts them into liters of $CO_{2}$. The data are stored and displayed in the Brewblox^®^ interface.

**Figure 1:**
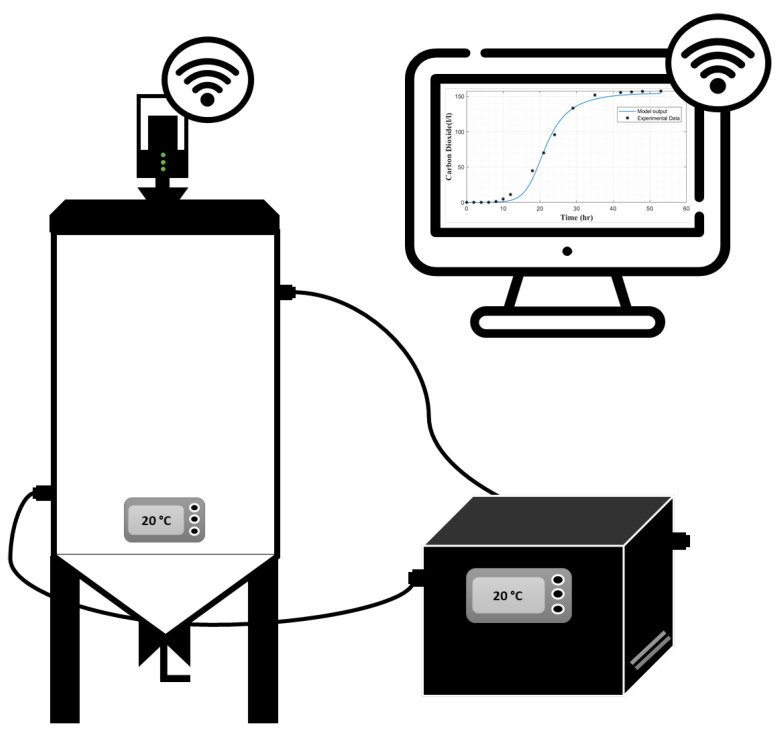
Stainless-steel fermenter pilot plant monitoring set-up.

Besides the two online probes, a CDR BeerLab^®^ analyzer is used to obtain offline measurements of sugar, ethanol, and vicinal diketone (VDK) concentrations. The scheme of the full experimental setup is displayed in Figure 1.

In this study, ale beer fermentation was considered and carried out at a temperature ranging from 17 to 26 °C. To obtain the wort, the malt is crushed using a mill. Indeed, the grain must only be broken, but not grounded. The next step is mashing, where sugars are obtained from starch. The crushed grain is added to a boiler tank (35 L, Grainfather^®^) containing 19 L of water at 48 °C. The mashing step consists of four stages at different temperatures and times described in Table 1. Once mashing is finished, the grain is rinsed with water at 75 °C until 24 L of wort is obtained. Usually, the quantity of added water is 8 L. Then, the wort is boiled to sterilize the liquid. The latter step is carried out for 80 min at 100 °C. Hop is added at 40 min and 65 min. Eventually, the wort is cooled down as fast as possible to the desired fermentation temperature with the help of a counter-flow wort cooler. The cold wort is transferred in the fermentation tank, filled up to 17 L.

A set of four isothermal batch fermentations without agitation are carried out using different operating conditions described in Table 2. Each experiment is carried out once, but two replicates are taken and analyzed for each sample. The total sampling volume represents less than 10% of the initial wort volume (17 L), a condition to neglect the volume changes. Samples are taken every 2 to 3 h during the first 36 h. After this period, the process enters a stationary phase and the sampling time is therefore adapted at irregular, longer, time intervals. To analyze the samples with the CDR BeerLab^®^, it is necessary to achieve preprocessing, including degasification and centrifugation to eliminate everything that could interfere during the measurement.

## 3. Mathematical Models of Beer Fermentation

Beer fermentation has been studied extensively, providing various mathematical models, and this section proposes a brief description of three of them, considered as milestones in the research field and used as a reference in the upcoming model development. These mathematical models can provide precious support to design the fermentation operating conditions.

### 3.1. The Model of Gee and Ramirez

This model published in 1994 includes the main components of fermentation, e.g., sugars, biomass, and ethanol, as well as amino acids, fusel alcohols, VDKs, and acetaldehydes, which impact the flavor, often in an undesirable way [7]. Esters also have an important role in the aroma and may add some pleasant character in moderate ranges, but undesired hard fruity tastes at higher levels.

The sugar uptake model reads as follows:

(1a) $$\begin{array}{cc} \frac{dG}{dt} & =-\mu_{G}X, \end{array}$$

(1b) $$\begin{array}{cc} \frac{dM}{dt} & =-\mu_{M}X, \end{array}$$

(1c) $$\begin{array}{cc} \frac{dN}{dt} & =-\mu_{N}X, \end{array}$$

where *G*, *M*, and *N*, respectively, stand for glucose, maltose, and maltotriose. The specific growth rates are built upon classical kinetic activation (Monod law) and inhibition factors and are given by:

(2a) $$\begin{array}{cc} \mu_{G} & =\frac{\mu_{G}G}{K_{G}+G}, \end{array}$$

(2b) $$\begin{array}{cc} \mu_{M} & =\frac{\mu_{M}M}{K_{M}+M}\frac{K_{G}^{\prime }}{K_{G}^{\prime }+G}, \end{array}$$

(2c) $$\begin{array}{cc} \mu_{N} & =\frac{\mu_{N}N}{K_{N}+N}\frac{K_{G}^{\prime }}{K_{G}^{\prime }+G}\frac{K_{M}^{\prime }}{K_{M}^{\prime }+M}, \end{array}$$

where $\mu_{i}$ are maximum rate constants (i=G,M,N), while $K_{i}$ and $K_{j}^{\prime }$ (j=G,M) are, respectively, the half-saturation and inhibition constants, all assumed to depend on the temperature following an Arrhenius law of the form:

(3) $$r=Aexp\left(\frac{B}{RT}\right),$$

where *A* and *B* are, respectively, the Arrhenius frequency factor and the activation energy. *R* is the ideal gas constant.

Biomass production is represented by the mass-balance ODE:

(4) $$\frac{dX}{dt}=\mu_{X}X,$$

where

(5) $$\mu_{x}=Y_{XG}\mu_{1}+Y_{XM}\mu_{2}+Y_{XN}\mu_{3}$$

is the biomass growth rate, a function of the several sugar intake rates. $Y_{Xi}$ (i=G,M,N) are the yield coefficients of biomass with respect to the specific sugars.

The ethanol concentration is assumed to evolve proportionally to the variations of the sugar concentrations, resulting in an algebraic equation of the form:

(6) $$E=E_{0}+Y_{EG}\left(G_{0}-G\right)+Y_{EM}\left(M_{0}-M\right)+Y_{EN}\left(N_{0}-N\right),$$

where $Y_{Ei}$ (i=G,M,N) are the yield coefficients of ethanol with respect to the consumed sugars.

Three main amino acids are considered, which are responsible for the formation of flavor compounds in the beer like the fusel alcohols. The amino acids are described by the following differential equations:

(7) $$\frac{d\xi }{dt}=-Y_{\xi }\;\frac{\xi }{K_{\xi }+\xi }\frac{dX}{dt}=-Y_{\xi }\mu_{X}\;\frac{\xi }{K_{\xi }+\xi }X,$$

where $Y_{\xi }$ and $K_{\xi }$ are, respectively, the yield coefficients and inhibition constants with species ξ= leucine (L), isoleucine (I), and valine (V).

The impact of fusel alcohols is a plastic, solvent-like flavor. Moreover, some experiments achieved in [11] have also linked higher alcohol levels with physiological effects associated with hangovers. The four fusel alcohols represented in the model are isobutyl alcohol (IB), isoamyl alcohol (IA), 2-methyl-1-butanol (MB), and n-propanol (P).

(8a) $$\begin{array}{cc} \frac{dIB}{dt} & =Y_{IB}\mu_{V}X, \end{array}$$

(8b) $$\begin{array}{cc} \frac{dIA}{dt} & =Y_{IA}\mu_{L}X, \end{array}$$

(8c) $$\begin{array}{cc} \frac{dMB}{dt} & =Y_{MB}\mu_{I}X, \end{array}$$

(8d) $$\begin{array}{cc} \frac{dP}{dt} & =Y_{P}\left(\mu_{V}+\mu_{I}\right)X, \end{array}$$

where $Y_{\zeta }$ (ζ=IB,IA,MB,P) are yield coefficients, and $\mu_{\xi }$ are specific rates expressed as $\mu_{\xi }=-\frac{1}{X}\;\frac{d\xi }{dt}=Y_{\xi }\mu_{X}\;\frac{\xi }{K_{\xi }+\xi }$(ξ=L,I,V).

Esters contribute mainly to the aroma of the beer due to their high volatility. In moderate concentrations, they can confer a pleasant character to the beer. However, once in excess, the aroma becomes overly fruity, which is undesired by most consumers. Principal esters are ethyl acetate (EA), ethyl caproate (EC), and isoamyl acetate (IAc).

(9a) $$\begin{array}{cc} \frac{dEA}{dt} & =Y_{EA}\left(\mu_{G}+\mu_{M}+\mu_{N}\right)X, \end{array}$$

(9b) $$\begin{array}{cc} \frac{dEC}{dt} & =Y_{EC}\mu_{X}X, \end{array}$$

(9c) $$\begin{array}{cc} \frac{dIAc}{dt} & =Y_{IAc}\mu_{IAc}X, \end{array}$$

where $Y_{\gamma }$ are the yield coefficients (γ=EA,EC,IAc) and $\mu_{IAC}$ is the maximum isoamyl acetate formation rate.

The common practice recommends completely removing vicinal diketones (VDKs) since they add some undesired buttery flavor notes. VDK production is assumed to be proportional to the growth rate, while their possible re-assimilation by yeast to form other by-products is proportional to their concentration. It must be noticed that acetaldehyde (AAl) is another compound showing similar behavior to the VDKs. The mass-balance equations read:

(10a) $$\begin{array}{cc} \frac{dVDK}{dt} & =Y_{VDK}\mu_{X}X-r_{VDK}VDKX, \end{array}$$

(10b) $$\begin{array}{cc} \frac{dAAl}{dt} & =Y_{AAl}\left(\mu_{G}+\mu_{M}+\mu_{N}\right)X-r_{AAl}AAlX, \end{array}$$

where $Y_{\omega }$ defines the yield coefficients and $r_{\omega }$ defines first-order rate constants (ω=VDK,AAl). $\mu_{X}$ is the biomass growth rate and $\mu_{G},\mu_{M},\mu_{N}$ are the sugar consumption rates.

This model therefore proposes a detailed description of the flavor and aroma of the beer but also presents the drawback of being difficult to apply in a realistic context since it requires numerous and often expensive advanced on-line monitoring devices. Indeed, in practice, biomass concentration is only measured at the beginning of a batch without further monitoring. Moreover, most of the parameters are temperature-dependent, either imposing rigorous operating conditions (i.e., only one constant temperature level) or parameter estimation of the temperature-dependent functions (which requires data collection at various temperatures).

### 3.2. Model of De Andrés-Toro et al.

This model, published in 1998, is more concise than the previous one as it only considers five state variables: sugars, biomass, ethanol, ethyl acetate, and diacetyl (i.e., vicinal diketones) [8]. Ethyl acetate and diacetyl are assumed to be the most influencing compounds regarding aroma and flavor. In the following, the model dynamics are described state-by-state. Biomass is segregated into three types: lagged, active, and dead. It is indeed assumed that part of the biomass goes through several states during the process, first in a lag phase when the fermentation starts, then in an active (growing) state, and eventually in an inactive (dead) state.

$$\begin{array}{c} \mathrm{Lagged}\mathrm{biomass} \end{array}$$

(11a) $$\begin{array}{cc} \frac{dX_{L}}{dt} & =-\mu_{L}X_{L},\\ \mathrm{Active}\mathrm{biomass} \end{array}$$

(11b) $$\begin{array}{cc} \frac{dX_{A}}{dt} & =\mu_{X}X_{A}+\mu_{L}X_{L}-\mu_{DT}X_{A},\\ \mathrm{Dead}\mathrm{biomass} \end{array}$$

(11c) $$\begin{array}{cc} \frac{dX_{D}}{dt} & =\mu_{DT}X_{A}-\mu_{SD}X_{D}, \end{array}$$

where the lagged biomass becomes active at the specific rate $\mu_{L}$, the active biomass grows at the specific rate $\mu_{X}$ and dies at the rate $\mu_{DT}$, while the dead biomass settles in the bottom of the reactor at the rate $\mu_{SD}$. $\mu_{X}$ and $\mu_{SD}$ are further defined as

(12) $$\begin{array}{cc} \mu_{X} & =\frac{\mu_{X_{0}}S}{0.5S_{0}+E}, \end{array}$$

(13) $$\begin{array}{cc} \mu_{SD} & =\frac{\mu_{SD_{0}}0.5S_{0}}{0.5S_{0}+E}. \end{array}$$

$\mu_{X}$ represents an activation by the substrate *S* and an inhibition by ethanol *E*. The inhibition constant is assumed to be inversely proportional to half the initial substrate concentration $S_{0}$ (indeed two units of S give one unit of E in the stoichiometry of the reaction). $\mu_{SD}$ describes an inhibition by ethanol, which is directly related to $CO_{2}$, which is not a variable in this model, but whose bubbles impair the settling phenomenon. The inhibition constant is again related to half the initial substrate concentration (i.e., maximal quantity of ethanol that can be produced).

Sugar consumption follows a Monod law according to:

(14) $$\frac{dS}{dt}=-\mu_{S}X_{A},$$

with

(15) $$\mu_{S}=\frac{\mu_{s_{0}}S}{K_{S}+S},$$

in which $\mu_{S_{0}}$ is the maximum specific consumption rate and $K_{S}$ is the half-saturation constant.

Ethanol production is described by

(16) $$\frac{dE}{dt}=\mu_{E}X_{A},$$

where the specific rate includes a Monod factor with respect to the substrate *S* and an inhibition factor related to the ethanol concentration. This factor vanishes when the ethanol reaches the maximum value $0.5S_{0}$:

(17) $$\mu_{E}=\frac{\mu_{E_{0}}S}{k_{E}+S}\left(1-\frac{E}{0.5S_{0}}\right).$$

Ethyl acetate is produced as a byproduct of active biomass growth:

(18) $$\frac{dEA}{dt}=Y_{EA}\mu_{X}X_{A},$$

where $Y_{EA}$ is the yield coefficient.

Diacetyl is a component belonging to the vicinal diketones (VDKs), which is produced as the biomass grows by consuming the sugars. Afterward, diacetyl is reduced into acetoin with a reduction rate $r_{VDK}$ activated in the presence of ethanol:

(19) $$\frac{dVDK}{dt}=k_{VDK}SX_{A}-r_{VDK}VDKE.$$

All the parameters are assumed to be affected by temperature according to the Arrhenius law (Equation (3)).

The biomass segregation model provides an accurate and consistent description of the process but, as a drawback, requires the corresponding monitoring equipment. In the study of [8], total biomass was measured online by absorbance change detection in a photocell, while the biomass state classification was made based on pre-established assumptions.

### 3.3. Model of Trelea et al.

The originality of the model of Trelea et al. [9], with respect to the previous ones, is that it considers the carbon dioxide dynamics instead of the biomass dynamics. Carbon dioxide sensors are indeed easily implemented and calibrated on-line, reliable, and significantly cheaper than biomass measurement devices.

The evolution of carbon dioxide is related to yeast growth, sugar consumption, and ethanol production. $CO_{2}$ dynamics are assumed to be driven by a Monod law describing sugar activation and saturation effects, an inhibition factor taking account of the decreasing cell respiratory capacity following ethanol accumulation. The influence of the initial biomass concentration on the initial $CO_{2}$ production rate is also taken into account:

(20) $$\frac{dCO_{2}}{dt}=\mu_{max}\frac{S}{K_{S}+S}\frac{1}{1+K_{I}E^{2}}\left(CO_{2}+C_{0}X_{0}\right),$$

where $K_{S}$ is the half-saturation coefficient, $K_{I}$ is the inhibition constant, and $C_{0}$ is a conversion factor.

Algebraic equations describe the evolution of the sugar and ethanol concentrations in relation to $CO_{2}$ as suggested in [12]:

(21) $$S=S_{0}-Y_{S}CO_{2},$$

(22) $$E=Y_{E}CO_{2},$$

where $Y_{S}$ and $Y_{E}$ are the corresponding yield coefficients. The main advantage of this model lies in its practical and control-oriented description of the fermentation process, considering a variable that can be easily measured, such as carbon dioxide. The maximum specific growth rate, $\mu_{max}$, is however, assumed to depend on several operating parameters, such as pressure, temperature, and initial yeast concentration, as follows:

(23) $$\mu_{max}=a_{T}T_{n}+a_{P}P_{n}+a_{X}X_{0,n}+a_{TP}T_{n}P_{n}+a_{TX}P_{n}X_{0,n}+a_{0},$$

where $a_{i}$ are parameters to be estimated.

### 3.4. Proposed Mathematical Models

In this study, two mathematical models of beer fermentation are proposed, one based on biomass dynamics and the other on $CO_{2}$ dynamics. These models take inspiration from the mathematical developments of the previous sections and attempt to describe experiments performed with the beer fermenter described in Section 2.

**Figure 2:**
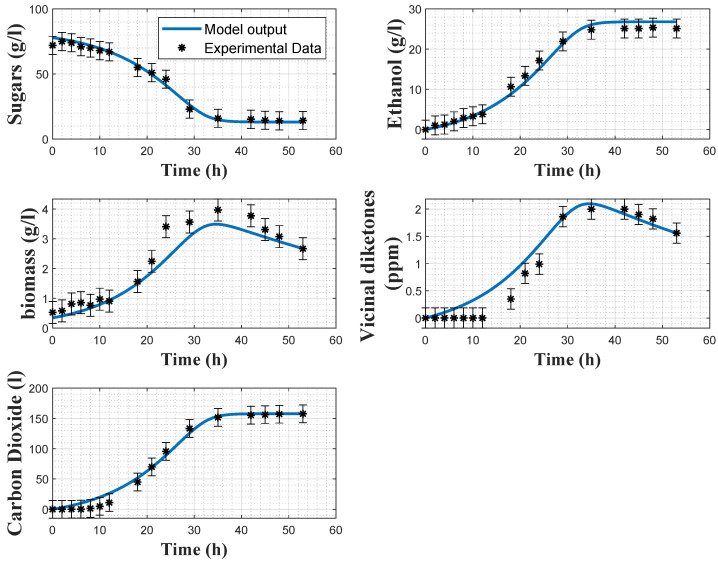
Direct validation of the biomass model with the data from experiment 2. Stars: experimental data. Error bars: 95% confidence intervals. Continuous blue line: model prediction.

Figure 2 shows some data collected in a batch experiment at 19 °C. It is apparent that sugar was not completely consumed at the end of the fermentation, with a residual concentration of about 12 g/L (this behavior was confirmed in repeated experiments in the same and different conditions). Two possible causes of this sluggish fermentation in the end of batch were explored in additional experiments, including biomass settling and water quality. However, gentle agitation and tests with different water sources did not influence the initial observation. Other causes such as the depletion of some components required for yeast proliferation and maintenance (such as nitrogen, sterols, fatty acids) could not be assessed. The models were, therefore, adapted to describe the experimental observations. As the published models only consider the total consumption of sugars, structural modifications were made to cope with this type of behaviour. The changes mostly impact the definition of the specific growth rate, as explained in the following sections.

#### 3.4.1. Dynamic Model Based on Biomass

An overall alcoholic fermentation reaction can be written as follows:

(24) $$k_{S}S\overset{r_{1}}{\to }k_{E}E+k_{V}\mathrm{VDK}+k_{CO_{2}}{CO}_{2}+X$$

where sugars *S* are consumed and converted by yeasts *X* into ethanol *E*, vicinal diketones VDK, and carbon dioxide $CO_{2}$. $k_{S}$, $k_{E}$, $k_{V}$, and $k_{{CO}_{2}}$ represent the yield coefficients of sugar, ethanol, vicinal diketones, and carbon dioxide, respectively.

Vicinal diketones are later reduced by yeasts, producing 2-3-butanodiol *P* in the following second reaction:

(25) $$\mathrm{VDK}\overset{r_{2}}{\to }P$$

From Equations (24) and (25), a set of mass-balance ODEs can be derived:

(26a) $$\begin{array}{cc} \frac{dX}{dt} & =\mu_{X}X-\delta_{X}X, \end{array}$$

(26b) $$\begin{array}{cc} \frac{dS}{dt} & =-k_{S}\mu_{X}X, \end{array}$$

(26c) $$\begin{array}{cc} \frac{dE}{dt} & =k_{E}\mu_{X}X \end{array}$$

(26d) $$\begin{array}{cc} \frac{dCO_{2}}{dt} & =-k_{CO_{2}}\mu_{X}X, \end{array}$$

(26e) $$\begin{array}{cc} \frac{dVDK}{dt} & =k_{V}\mu_{X}X-r_{VDK}VDK. \end{array}$$

The specific growth rate is defined as:

(27a) $$\begin{array}{cc} \mu_{X} & =\mu_{max}\left(1-\frac{S_{min}}{S}\right),\;for\;S\geq S_{min}, \end{array}$$

(27b) $$\begin{array}{cc} \; & =0,\;\;for\;S\leq S_{min}. \end{array}$$

The specific growth rate usually represented by a Monod law is replaced by a Droop-like factor [13], commonly used to describe microalgae growth. This kinetic structure expresses that a minimum level of sugar $S_{min}$ is necessary to trigger growth. Above this threshold, the Monod factor has an activation/saturation effect similar to a Monod factor. Furthermore, a decrease in biomass is observed due to settling and is represented by the coefficient $\delta_{X}$ in Equation (26a).

In classical batch fermentation, the temperature is the only variable that can be manipulated during the batch to control the evolution of the fermentation. Based on the previously discussed modeling studies [7,8,9] and experimental observation, a temperature-dependency of the maximum specific growth rate $\mu_{max}$ is expected that can be described following several structural laws [14]. Moreover, the reduction speed of the VDKs, $r_{VDK}$, is also affected by temperature. In Table A1, the definitions and units of each parameter are listed.

#### 3.4.2. Dynamic Model Based on Carbon Dioxide

The yeast concentration is difficult to monitor during the batch and, most of the time, this variable is either indirectly measured by the turbidity of the wort, or simply by sampling and cell counting using a cytometer or a dry-weight method. Hence, there is a strong motivation to monitor other, more accessible, process variables and develop dynamic models describing their evolution. Carbon dioxide may be such an indicator since it is a product of the biochemical reactions related to sugar oxidation, which provides the necessary energy to the yeast cells to grow and leads to ethanol production if sugar is in excess, activating the overflow metabolism [15,16]. In this study, the model of [9] was adapted in the following way:

(28a) $$\begin{array}{cc} \frac{d{CO}_{2}}{dt} & =\mu_{X}{CO}_{2}, \end{array}$$

(28b) $$\begin{array}{cc} S & =S_{0}-k_{S}{CO}_{2}, \end{array}$$

(28c) $$\begin{array}{cc} E & =E_{0}+k_{E}{CO}_{2}, \end{array}$$

(28d) $$\begin{array}{cc} \frac{dVDK}{dt} & =k_{V}\mu_{X}{CO}_{2}-r_{VDK}VDK, \end{array}$$

where the specific growth rate is given by:

(29) $$\mu_{x}=\mu_{max}\frac{S}{K_{S}+S}\left(1-\frac{{CO}_{2}}{Cp_{max}S_{0}}\right).$$

The kinetic description contains a Monod law for the sugar activation and saturation effects, and a logistic factor to mimic the observed sigmoidal evolution of carbon dioxide. The data sets also reveal that the maximum production of carbon dioxide is correlated with the initial sugar concentration $S_{0}$, which therefore defines the maximum $CO_{2}$ level (i.e., the carrying capacity of the logistic model). The initial biomass concentration, which is assumed to be known (measured) and directly correlated to the $CO_{2}$ dynamics in [9], was not used in the current study since the initial condition of $CO_{2}$ was available. The rates $\mu_{max}$ and $r_{VDK}$ are assumed to depend on temperature and Table A2 lists the definitions and units of some parameters.

## 4. Structural Identifiability and Observability of the Models

Identifiability globally refers to the possibility of identifying the model parameters from the available data. A model is structurally identifiable if all the parameters can be uniquely determined from ideal measurements of its outputs, i.e., collected in continuous time without errors or noise, and the knowledge of the dynamic equations [17]. If this property is not met, any further effort to estimate the non-identifiable parameters will be vain. However, the identifiability analysis is often omitted due to the assumed complexity of the mathematical developments required to achieve the analysis. Recently, several methodologies and toolboxes have been developed to significantly ease the task, as reviewed in [18]. Some of these software tools are DAISY [19], GENSSI [20], STRIKE-GOLDD [10], and SIAN [21].

On the other hand, practical identifiability deals with the possibility of assessing all or some of the model parameters under realistic conditions, e.g., sampled data and measurement noise. The Fisher Information Matrix (FIM) is useful to assess practical identifiability through a rank test condition. An ill-conditioned FIM can indicate poor practical parameter identifiability even if structural identifiability is met.

In this work, STRIKE-GOLDD (STRuctural Identifiability taken as Extended-Generalized Observability with Lie Derivatives and Decomposition) was used to investigate the structural identifiability of the proposed beer fermentation models. This software tool has been developed in MATLAB^®^ and addresses identifiability based on the concept of observability. To this end, the model is extended by considering its model parameters as state variables with zero dynamics. The results obtained for both models indicate that structural identifiability is ensured only when all the state variables are measured.

Another property of interest is observability, which is a prerequisite to the design of a state observer to reconstruct nonmeasured state variables. The results of the analysis are provided in Table 3 and Table 4 for the two dynamic models. For the model based on biomass, the analysis reveals that the set of three measurements $\left[{CO}_{2},E,VDK\right]$ is necessary to guarantee observability. The set $\left[{CO}_{2},E,S\right]$ shows partial observability as VDK cannot be reconstructed but biomass *X* could. The carbon dioxide model requires the measurement of VDK together with another variable ($CO_{2}$ or *E* or *S*) to fulfill the observability condition. An observer could, therefore, be designed to estimate the sugar concentration online, which is the most expensive measurement using an online or at-line hardware probe.

## 5. Parameter Identification Problem

Parameter identification is achieved using classical nonlinear parameter estimation techniques [22]. The procedure considers a Weighted Least-Square (WLS) criterion, i.e., a weighted sum of squared differences between model predictions and experimental measurements:

(30) $$J\left(\theta \right)=\sum_{i=1}^{M}\left(y\left(t_{i}\right)-y_{model}\left(t_{i},\theta \right){)}^{T}W^{-1}\left(y\left(t_{i}\right)-y_{model}\left(t_{i},\theta \right)\right)\right)$$

where J is the value of the cost function, $y(t_{i})$ is the vector of *N* measured variables at the measurement instant $t_{i}$ (i=1,…,M), $y_{model}\left(t_{i},\theta \right)$ is the model prediction that depends on the set of *P* parameters θ to be identified, and W is a normalization matrix where the diagonal elements are chosen as the squares of the maximum measurements values of each component concentration. This choice allows normalization of the prediction errors, and is particularly well-suited to a relative error model where it is assumed that the error is proportional to the maximum values of the observed variables:

(31) $$W=\left[\begin{array}{cccc} \max_{t_{i}}\left(y_{1}^{2}\right) & 0 & \cdots & 0\\ 0 & \max_{t_{i}}{\left(y_{2}\right)}^{2} & \cdots & 0\\ \vdots & \vdots & \ddots & \vdots \\ 0 & 0 & \cdots & \max_{t_{i}}{\left(y_{N}\right)}^{2} \end{array}\right]$$

The estimated parameter set is obtained by minimizing a cost function J(θ) as follows:

(32) $$\hat{\theta }=\arg\min_{\theta }J\left(\theta \right)$$

To achieve the minimization of (32), a two-step procedure is implemented: (a) a multi-start strategy defines random sets of initial parameter values to cover as much as possible of the search field. The minimization of (32) is first performed using the Matlab^®^ optimizer fminsearch (Nelder-Mead algorithm); (b) the Matlab lsqnonlin optimizer is subsequently used from the identified global minimum (i.e., the smallest local minimum identified in the search space by fminsearch) to refine the minimization and compute the Jacobian matrix containing the model parameter sensitivities, denoted $y_{\theta }$. These sensitivities can be exploited to compute the Fisher Information Matrix (FIM) defined as:

(33) $$\mathrm{FIM}=\sum_{i=1}^{M}y_{\theta }^{T}\left(t_{i}\right){\hat{\Omega }}^{-1}y_{\theta }\left(t_{i}\right)$$

where $\hat{\Omega }={\hat{\epsilon }}^{2}W$ is the a posteriori covariance matrix of the measurement errors, which can be evaluated using the weighting matrix W (Equation (31)) and an a posteriori estimator of the relative measurement error:

(34) $${\hat{\epsilon }}^{2}=\frac{J^{*}}{MN-P}$$

where $J^{*}$ is the value of the cost function at the optimum, MN represents the total number of data, and *P* is the number of estimated parameters θ. An estimate of the parameter estimation error covariance matrix can then be inferred from the Cramer–Rao bound as follows:

(35) $$\hat{\Sigma }={\mathrm{FIM}}^{-1}$$

From the diagonal of the covariance matrix $\hat{\Sigma }$, the standard deviations for each parameter can be extracted and the corresponding coefficients of variations can be calculated as:

(36) $$\mathrm{CV}=\frac{\sigma }{{\hat{\theta }}_{i}}$$

To achieve the estimation of the parameters of the beer fermentation models, a total of 4 batch experiments are considered as shown in Table 2. Out of these 4 experiments, 3 are used for parameter estimation and model direct validation (experiments 2 to 4), while experiment 1 is used for cross-validation. An important point of the current work is the use of all the samples of experiments 2, 3, 4 to achieve the identification, including two temperature-varying parameters, i.e., the specific growth rate $\mu_{max}$ and the VDK reduction rate $r_{VDK}$. Previous studies have indeed demonstrated that the other parameters do not change significantly with temperature. In addition to the stoichiometric and kinetic parameters, the initial conditions are also considered unknown (and are therefore estimated) since possibly corrupted by measurement noise.

### 5.1. Biomass Model

This model counts 8 parameters ($k_{S}$, $k_{E}$, $S_{min}$, $g_{x}$, $k_{V}$, $k_{{CO}_{2}}$, $\mu_{max}$, $r_{a}$) to be estimated. The dependence on temperature of the parameters $\mu_{max}$ and $r_{VDK}$, is formulated as follows:

(37) $$\mu_{max}=a\ln\left(T\right)+b;\;\;\;\;\;\;\;\;r_{VDK}=c\ln\left(T\right)+d;$$

introducing the additional parameters *a*, *b*, *c*, *d*. Several nonlinear model structures have been considered to correlate the parameters with the temperature. It turned out that the selected logarithmic structure provides the best results.

In practice, the identification proceeds in three steps: (a) a first parameter estimation without explicit temperature dependence (i.e., $\mu_{max}$ and $r_{VDK}$ are considered constant), (b) an estimation of the four parameters linked to the temperature dependence (the others being fixed at their previously estimated values), and (c) a global identification of all the parameters starting from the previous estimates.

Good practice recommends partitioning the data using a ratio of approximately 75/25 for parameter estimation (and subsequent direct validation), and cross-validation, respectively. Accordingly, three experimental data sets are used in direct validation and the remaining one in cross-validation. Since the parameter estimation procedure aims at capturing information on the process in a wide range of operations, it is legitimate to include experiments with different initial sugar and biomass concentrations and temperature levels. Particularly, it is important to collect informative data regarding the evolution of $\mu_{max}$ and $r_{VDK}$ with respect to temperature in (37). Among the several possible data partitions, one possible combination appears to be: experiments 2, 3, 4 for parameter estimation (and direct validation) and experiment 1 for cross-validation. Indeed, experiments 1 and 2 are carried out at the same temperature, but experiment 2 also includes different sugar and biomass initial conditions. Table 5 reports the values of the estimated parameters and their coefficients of variations.

**Figure 3:**
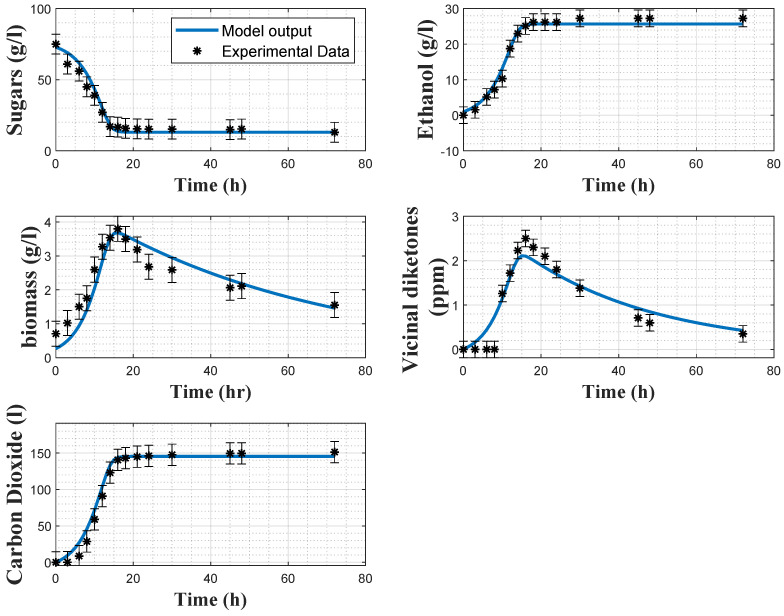
Direct validation of the biomass model with the data from experiment 4. Stars: experimental data. Error bars: 95% confidence intervals. Continuous blue line: model prediction.

Figure 2 and Figure 3 show some direct validation results, i.e., the fitting of the model to the experimental data collected in experiments 2 and 4 together with the a posteriori error bars on the experimental data. The model reproduces quite well the dynamics of the several variables, even if the biomass predictions sometimes deviate from the confidence intervals of the data, and some deviations in the VDK production are also observed in the early hours. The coefficients of variations confirm the good estimation results, as the maximum relative CV is 11% for the minimum substrate quota $S_{min}$.

**Figure 4:**
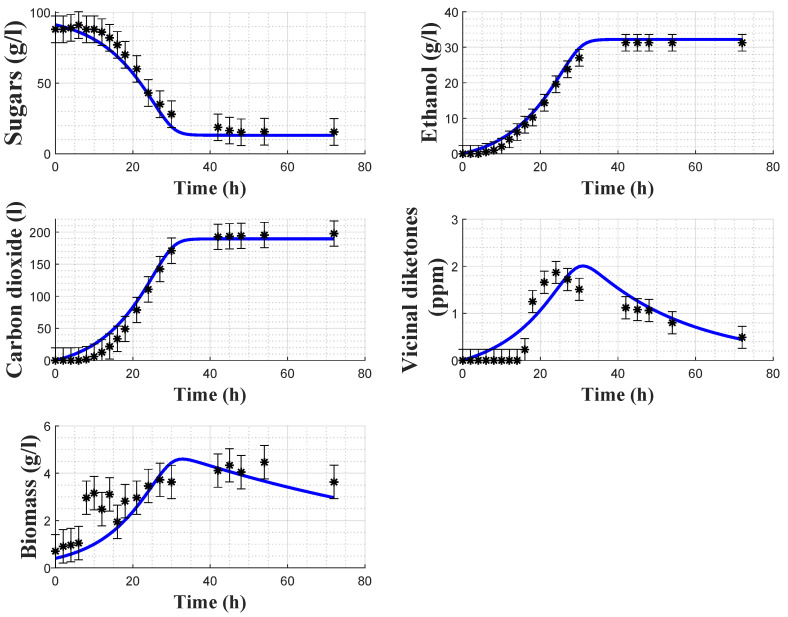
Cross-validation of the biomass model with the data from experiment 1. Stars: experimental data. Error bars: 95% confidence intervals. Continuous blue line: model prediction.

In order to assess the model predictive capacity, cross-validation is achieved using the dataset from experiment 1. In this case, only the initial conditions are estimated while the parameters are kept fixed. As shown in Figure 4, the model predicts satisfactorily the experimental data. The biomass data again has some uncertainty, which can probably be linked to several factors such as cell counting errors, biomass mixing (to counteract biomass settling and collect representative samples) and nitrogen limitation [23].

Vicinal diketone dynamics present a varying latency phase followed by production/consumption, both driven by biomass dynamics. The uncertainty on the latency period compromises the resulting fitting since biomass does not exhibit the same behavior in the early phase of fermentation.

### 5.2. Carbon Dioxide Model

A similar procedure was applied to estimate the values of the carbon dioxide model parameters. In this case, the dependence on temperature of $\mu_{max}$ and $r_{V}DK$ is best represented by:

(38) $$\mu_{max}=a\ln\left(T\right)+b;\;\;\;\;\;\;\;\;r_{VDK}=cT^{2}+dT+e;$$

Hence, the resulting model counts 10 parameters ($k_{S}$, $k_{E}$, $K_{S}$, $Cp_{max}$, $k_{V}$, *a*, *b*, *c*, *d*, *e*), and Table 6 reports the estimated values with their respective coefficients of variations. As can be noticed, the latter are smaller than the ones of the previous model, mainly due to the absence of biomass measurement and the associated uncertainty.

The identification is again decomposed into distinctive steps: (a) estimation of the parameters without temperature dependence and with an arbitrary value for $K_{S}$ whose practical identifiability is poor, (b) estimation of $K_{S}$ with all the other parameters fixed at their previously estimated values, (c) estimation of the parameter linked to the temperature dependence (a to e) with all the others fixed to their previous values, and (d) final re-estimation of all the parameters.

**Figure 5:**
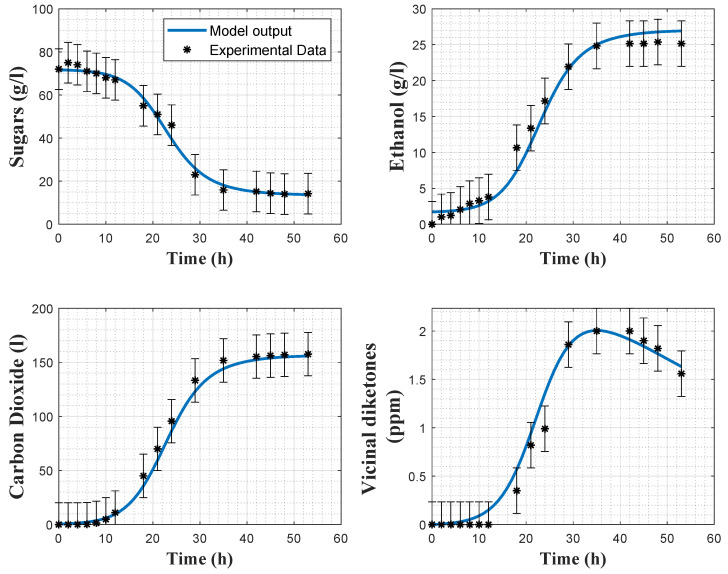
Direct validation of the carbon dioxide model with the data from experiment 2. Stars: experimental data. Error bars: 95% confidence intervals. Continuous blue line: model prediction.

**Figure 6:**
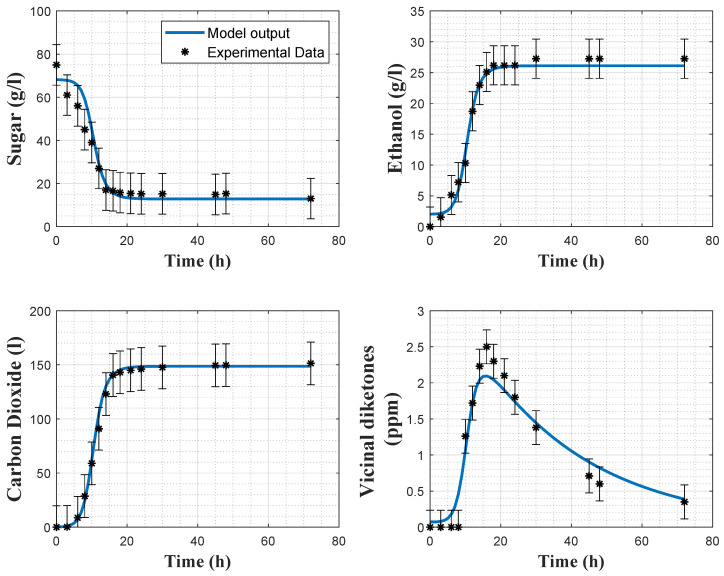
Direct validation of the carbon dioxide model with the data from experiment 4. Stars: experimental data. Error bars: 95% confidence intervals. Continuous blue line: model prediction.

Figure 5 and Figure 6 show the direct validation with experiments 2 and 4, as well as the a posteriori error bars on the experimental data.

**Figure 7:**
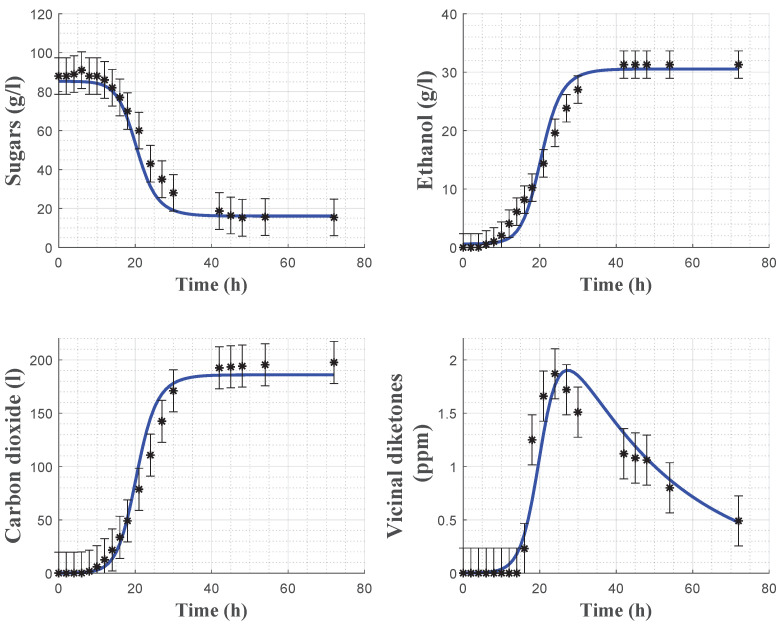
Cross-validation of the carbon dioxide model with the data from experiment 1. Stars: experimental data. Error bars: 95% confidence intervals. Continuous blue line: model prediction.

Model cross-validation using experiment 1 is shown in Figure 7 and confirms the satisfactory predictive capacity of the model, except for some minor deviations in the evolution of the VDKs due to the presence of a time-varying latency phase.

In Table 7, the root means square errors (RMSEs) are provided for each experiment and each variable separately. The cost function residuals of the direct validations are also provided. It can be observed that, overall, the RMSE values are small for both models. Regarding the biomass model, *X* and VDK present larger RMSEs than the other variables, due to the observed deviations between the model prediction and the experimental data in Figure 2 and Figure 3. Regarding the carbon dioxide model, RMSEs indicate a better fit with the experimental data. This statement is confirmed by Figure 5 and Figure 6. Cross-validation results also support this analysis since the RMSEs of the biomass and carbon dioxide models are, respectively, 1.997 and 0.846.

Discriminating among the proposed models is difficult since they target different variables. However, taking into account the cost function residuals, the carbon dioxide model fit better to the current operating conditions and monitoring set-up (J = 0.72) than the biomass model (J = 1.76). Furthermore, from a practical point of view, the identification of the dioxide carbon model requires a sensor configuration that is easier to set up, limiting offline analytical analysis. Conversely, the identification of the biomass model requires offline cell counting to measure yeast concentration. Moreover, considering process control, carbon dioxide online sensors are affordable, whereas biomass sensors are expensive (alternatively a biomass software sensor could be developed based on the measurements of $CO_{2}$, E, VDK). The main advantage of the biomass model lies in the provided information about the biomass metabolic state during the fermentation process, allowing a more straightforward detection of possible contamination.

## 6. Conclusions

The demand for processes with more rigorous quality standards, as is the case in the pharmaceutical industry, has led to the development of approaches such as process analytical technologies (PAT), now being extended to the agro-food sector and, more specifically, to the brewing industry. This work is motivated by the growing importance of mathematical modeling, in the context of PAT, to design process digital twins that can support lab-scale operations. Model-based advanced monitoring and control techniques can indeed be developed in view of optimizing and improving the process. In this study, two alternative models, initially proposed in seminal works, are adapted and identified under realistic experimental conditions. One of the models is based on the description of the biomass evolution, while the other, more pragmatic, considers carbon dioxide, a more accessible variable that can be measured with cheap sensors. These models take account of the temperature influence in a simple way. A systematic identification procedure is described. Cross-validation highlights the good predictive capability of both models, which are good candidates for model-based control.

## Figures and Tables

**Table 1:** Operating conditions of the scheduled mash steps: temperatures and times.

Step	Temperature (°C)	Time (min)
1	48	10
2	62	30
3	71	20
4	78	5

**Table 2:** Operating conditions of each fermentation batch: duration, temperature, and initial sugar and biomass concentrations.

Experiment	Time (h)	Temperature (°C)	S_0_ (g/L)	X_0_ (g/L)
1	72	19	88	7.05 × 10${}^{-1}$
2	54	19	72	5.29 × 10${}^{-1}$
3	96	21	75	7.05 × 10${}^{-1}$
4	72	28	81	7.05 × 10${}^{-1}$

**Table 3:** Observability analysis of the biomass model for several measurement configurations.

Measured Outputs	Observable
[$CO_{2}$, S]	No
[$CO_{2}$, E]	No
[S, E]	No
[$CO_{2}$, S, X]	No
[$CO_{2}$, E, VDK]	Yes
[$CO_{2}$, E, S]	VDK: No X: Yes
[$CO_{2}$, S, X, VDK]	No

**Table 4:** Observability analysis of the carbon dioxide model for several measurement configurations.

Measured Outputs	Observable
[$CO_{2}$, S]	No
[$CO_{2}$, E]	No
[S, E]	No
[S, VDK]	Yes
[E, VDK]	Yes
[$CO_{2}$, VDK]	Yes

**Table 5:** Parameter estimate values and coefficients of variation (CV) for the biomass model.

Parameter	Units	Value	CV (%)
$k_{S}$	gS/gX	15.3	7
$k_{E}$	gE/gX	6.31	6
$S_{min}$	g/L	13.1	11
$g_{x}$	h${}^{-1}$	1.67 × 10${}^{-2}$	7
$k_{V}$	gVDK/gX	6.51 × 10${}^{-1}$	10
$k_{{CO}_{2}}$	L	37.1	5
*a*	h${}^{-1}$	4.00 × 10${}^{-1}$	3
*b*	h${}^{-1}$	−1.1	5
*c*	h${}^{-1}$	2.50 × 10${}^{-2}$	10
*d*	h${}^{-1}$	−5.60 × 10${}^{-2}$	9

**Table 6:** Parameter estimate values and coefficients of variations (CV) for the carbon dioxide model.

Parameter	Units	Value	CV (%)
$k_{S}$	gS/lCO${}_{2}$	3.72 × 10${}^{-1}$	4
$k_{E}$	gE/lCO${}_{2}$	1.62 × 10${}^{-1}$	3
$K_{S}$	g/L,	12.0	9
$Cp_{max}$	L/g	2.18	3
$k_{V}$	ppm	1.74 × 10${}^{-2}$	4
*a*	h${}^{-1}$	1.41	6
*b*	h${}^{-1}$	− 3.89	8
*c*	°C${}^{-2}\;$h${}^{-1}$	− 3 × 10${}^{-4}$	12
*d*	°C${}^{-1}$ h${}^{-1}$	1.6 × 10${}^{-2}$	6
*e*	h${}^{-1}$	− 1.75 × 10${}^{-1}$	5

**Table 7:** Cost function residuals and relative RMSEs for each state variable of the two models, resulting from direct validation.

Model	Cost Function Residual	Variable	Exp 2 RMSE	Exp 3 RMSE	Exp 4 RMSE	Global RMSE
		$CO_{2}$	4.86 × 10${}^{-2}$	5.40 × 10${}^{-2}$	6.40 × 10${}^{-2}$	5.32 × 10${}^{-2}$
		*S*	3.37 × 10${}^{-2}$	8.12 × 10${}^{-2}$	5.43 × 10${}^{-2}$	5.80 × 10${}^{-2}$
Biomass	1.76	*E*	4.78 × 10${}^{-2}$	6.67 × 10${}^{-2}$	5.15 × 10${}^{-2}$	5.30 × 10${}^{-2}$
		*X*	9.96 × 10${}^{-2}$	9.63 × 10${}^{-2}$	1.62 × 10${}^{-1}$	1.09 × 10${}^{-1}$
		VDK	1.12 × 10${}^{-1}$	1.16 × 10${}^{-1}$	1.51 × 10${}^{-1}$	1.12 × 10${}^{-1}$
		$CO_{2}$	3.45 × 10${}^{-2}$	2.99 × 10${}^{-2}$	4.74 × 10${}^{-2}$	3.56 × 10${}^{-2}$
		*S*	2.23 × 10${}^{-2}$	7.40 × 10${}^{-2}$	4.63 × 10${}^{-2}$	5.36 × 10${}^{-2}$
Carbon dioxide	0.72	*E*	5.47 × 10${}^{-2}$	9.91 × 10${}^{-2}$	4.11 × 10${}^{-2}$	6.23 × 10${}^{-2}$
		VDK	6.26 × 10${}^{-2}$	8.59 × 10${}^{-2}$	1.12 × 10${}^{-1}$	7.50 × 10${}^{-2}$

## Data Availability

The data presented in this study are available on request from the corresponding author.

## Appendix A

**Table A1:** Parameter nomenclature list for biomass model.

Parameter	Definition	Units
$k_{S}$	Sugar yield coefficient	gS/gX
$k_{E}$	Ethanol yield coefficient	gE/gX
$S_{min}$	Minimum sugar quota	g/L
$\delta_{X}$	Settling constant	h${}^{-1}$
$k_{V}$	VDK yield coefficient	gVDK/gX
$k_{{CO}_{2}}$	$CO_{2}$ yield coefficient	L
*a*	Temperature-dependency coefficient	h${}^{-1}$
*b*	Temperature-dependency coefficient	h${}^{-1}$
*c*	Temperature-dependency coefficient	h${}^{-1}$
*d*	Temperature-dependency coefficient	h${}^{-1}$

**Table A2:** Parameter nomenclature list for carbon dioxide model.

Parameter	Definition	Units
$k_{S}$	Sugar yield coefficient	gS/l$CO_{2}$
$k_{E}$	Ethanol yield coefficient	gE/l$CO_{2}$
$K_{S}$	Substrate limitation coefficient	g/L
$Cp_{max}$	Carrying capacity	L/g
$k_{V}$	VDK yield coefficient	ppm
$k_{{CO}_{2}}$	$CO_{2}$ yield coefficient	L
*a*	Temperature-dependency coefficient	h${}^{-1}$
*b*	Temperature-dependency coefficient	h${}^{-1}$
*c*	Temperature-dependency coefficient	°C${}^{-2}$ h${}^{-1}$
*d*	Temperature-dependency coefficient	°C${}^{-1}$ h${}^{-1}$
*e*	Temperature-dependency coefficient	h${}^{-1}$
